# Management von SARD-ILD im Wandel: Aktuelle Herausforderungen und neue Therapieoptionen mit Rheuma-assoziierten interstitiellen Lungenerkrankungen

**DOI:** 10.1007/s00393-026-01807-3

**Published:** 2026-03-25

**Authors:** Torsten Witte, Jonas Schupp

**Affiliations:** 1 Klinik für Rheumatologie und Immunologie, Medizinische Hochschule Hannover, Carl-Neuberg-Str. 1, 30625 Hannover, Deutschland; 2 Klinik für Pneumologie und Infektiologie, Medizinische Hochschule Hannover, Carl-Neuberg-Str. 1, 30625 Hannover, Deutschland

**Keywords:** Rheumatische Systemerkrankungen, Interstitielle Lungenerkrankung, Progrediente pulmonale Fibrose, Therapie, Innovative Wirkstoffe, Systemic rheumatic diseases, Interstitial lung disease, Progressive pulmonary fibrosis, Therapy, Innovative agents

## Abstract

**Hintergrund:**

Rheumatische Systemerkrankungen mit interstitieller Lungenerkrankung (SARD-ILD) sind oft mit einer progredienten pulmonalen Fibrose (PPF) assoziiert, die durch eine zunehmende respiratorische Symptomatik, einen fortschreitenden Verlust der Lungenfunktion und eine ungünstige Prognose charakterisiert ist. Derzeit verfügbare Therapien wie Glukokortikoide, Immunsuppressiva und Antifibrotika können den Krankheitsverlauf je nach zugrunde liegender SARD-ILD häufig nur begrenzt beeinflussen, zudem schränken Nebenwirkungen die Therapie ein. Es besteht daher ein erheblicher Bedarf an wirksamen und gut verträglichen Behandlungsoptionen.

**Ziel der Arbeit:**

Dargestellt werden aktuelle Entwicklungen und neue Therapieansätze bei SARD-ILD und PPF sowie deren potenzielle Bedeutung für die klinische Praxis.

**Material und Methoden:**

Eine systematische PubMed-Recherche (Publikationen bis Mitte 2025) zu den Begriffen „SARD-ILD“, „CTD-ILD“, „PPF“ und „Treatment“ identifizierte relevante Studien zu neuen Behandlungsansätzen.

**Ergebnisse:**

Zu den derzeit in klinischer Entwicklung befindlichen Substanzen zählen u. a. Nerandomilast, Belimumab, Admilparant, Anifrolumab sowie inhalatives Treprostinil. Nerandomilast, ein präferentieller PDE4B-Inhibitor mit immunmodulatorischen, vaskulären und antifibrotischen Eigenschaften, reduzierte in der Phase-3-Studie FIBRONEER-ILD den Lungenfunktionsverlust und das Mortalitätsrisiko bei guter Verträglichkeit und könnte damit einen Wendepunkt im therapeutischen Management autoimmunassoziierter ILD darstellen.

**Diskussion:**

Innovative therapeutische Perspektiven für die SARD-ILD und PPF sind durch erfolgversprechende Arzneimittelstudien gegeben und könnten in Zukunft die Prognose und erhebliche Krankheitslast der Betroffenen merklich verbessern.

## Abstract (en)

**Background:**

Systemic rheumatic diseases with interstitial lung disease (SARD-ILD) are often associated with progressive pulmonary fibrosis (PPF), characterised by increasing respiratory symptoms, progressive loss of lung function, and an unfavourable prognosis. Currently available therapies, including glucocorticoids, immunosuppressants and antifibrotic agents, often have limited efficacy depending on the underlying SARD-ILD, while adverse events limit their use. Therefore, there is a substantial unmet need for effective and well-tolerated treatment options.

**Objectives:**

This review summarises current developments and emerging therapeutic approaches in SARD-ILD and PPF, highlighting their potential relevance for clinical practice.

**Materials and methods:**

A systematic PubMed search (publications up to mid-2025) using the terms “SARD-ILD”, “CTD-ILD”, “PPF” and “treatment” identified relevant studies investigating novel therapeutic strategies.

**Results:**

Substances currently under clinical investigation include nerandomilast, belimumab, admilparant, anifrolumab and inhaled treprostinil. Nerandomilast, a preferential PDE4B inhibitor with antifibrotic and anti-inflammatory properties, demonstrated a reduction in lung function loss and mortality risk with good tolerability in the phase‑3 FIBRONEER-ILD study, potentially representing a turning point in the management of autoimmune-associated ILD.

**Conclusion:**

Promising results from ongoing clinical studies provide new therapeutic perspectives for SARD-ILD and PPF and may significantly improve prognosis and disease burden in the future.

Rheumatische Systemerkrankungen können neben dem Bewegungsapparat auch zahlreiche innere Organe betreffen. Dabei gilt die pulmonale Beteiligung in Form einer interstitiellen Lungenerkrankung („systemic autoimmune rheumatic disease associated-interstitial lung disease“, SARD-ILD) als häufig und prognostisch relevant.

## Hintergrund

### Krankheitsbild und Prognose

Die SARD-ILD tritt vor allem bei systemischer Sklerose (SSc), idiopathischen inflammatorischen Myopathien (IIM), Sjögren-Syndrom (SjS) und rheumatoider Arthritis (RA) auf, kann jedoch grundsätzlich im Rahmen aller rheumatischen Systemerkrankungen entstehen [3]. SARD-ILD weisen einen heterogenen Verlauf auf. Rund 45 % der Patient:innen entwickeln eine progrediente pulmonale Fibrose (PPF), die durch zunehmende respiratorische Symptomatik, fortschreitende Einschränkung der Lungenfunktion und radiologisch nachweisbare Progression charakterisiert ist [6, 20]. Typische Symptome sind Belastungsdyspnoe, chronischer Husten und Müdigkeit, welche die Lebensqualität deutlich beeinträchtigen. SARD-ILD, insbesondere SSc-ILD, gehen vor allem bei progredientem Verlauf mit einer höheren Mortalität einher als rheumatische Erkrankungen ohne pulmonale Beteiligung [18]. Bei Patient:innen mit SARD-ILD und PPF ist die mediane Überlebenszeit im Vergleich zur SARD-ILD-Gesamtpopulation etwa halbiert [13].

### Derzeitige Therapie

Die Behandlung der SARD-ILD ist komplex und erfordert ein frühzeitiges Management durch Rheumatologie und Pneumologie, um eine Progression zu verzögern. Zu den therapeutischen Ansätzen zählen neben medikamentösen auch supportive Maßnahmen wie Impfungen, Sauerstofftherapie, pulmonale Rehabilitation und Rauchstopp. Bei fortgeschrittenen Erkrankungsverläufen können zudem eine Lungentransplantation oder, abhängig von der Grunderkrankung, zelluläre Therapien wie eine autologe hämatopoetische Stammzelltransplantation oder eine CAR-T-Zell-Therapie erwogen werden.

Die medikamentösen Therapieoptionen umfassen Glukokortikoide (z. B. Prednisolon), Immunsuppressiva (z. B. Cyclophosphamid [CYC], Mycophenolsäure, Azathioprin [AZA], Methotrexat [MTX], Cyclosporin, Tacrolimus), Antifibrotika (Nintedanib, Pirfenidon) sowie zielgerichtete Therapien einschließlich Januskinase(JAK)-Inhibitoren und Biologika (z. B. Tocilizumab [TCZ], Rituximab [RTX]). Mangels großer randomisierter Studien (RCTs) werden viele dieser Therapien off-label eingesetzt.

Die 2025 aktualisierten ERS/EULAR-Leitlinien geben evidenzbasierte Empfehlungen zur Therapie von SARD-ILD unter Berücksichtigung patientenspezifischer Risikofaktoren, extrapulmonaler Manifestationen, potenzieller Nebenwirkungen sowie des ILD-Schweregrads [2].

Bei SSc-ILD werden entzündliche Prozesse bevorzugt mit Immunsuppressiva wie Mycophenolsäure, CYC und RTX behandelt; während TCZ insbesondere bei früher diffus kutaner, entzündlicher SSc oder rasch progredienter Hautfibrose erwogen werden kann. Fibrotische Prozesse werden durch Antifibrotika wie Nintedanib adressiert, das in SSc-ILD auch ohne dokumentierte Progression eingesetzt werden kann. Auch bei RA-ILD steht die Entzündungshemmung, z. B. mit Abatacept und RTX, im Vordergrund, während bei Patient:innen mit einem UIP(„usual interstitial pneumonia“)-Muster zusätzlich Antifibrotika wie Nintedanib oder Pirfenidon in Betracht gezogen werden können. Bei IIM-ILD stellt die entzündungshemmende Therapie, meist kombiniert mit Glukokortikoiden, den Therapiestandard dar, wobei antifibrotische Maßnahmen bei vorliegender PPF ergänzend erwogen werden können. Gleiches gilt für weitere SARD-ILD, bei denen primär die Entzündung behandelt wird, während Antifibrotika bei PPF hinzugezogen werden können.

In der Regel ist bei allen SARD-ILD mit PPF eine Kombination aus Immunsuppressiva und Antifibrotika erforderlich, um sowohl die entzündlichen als auch die fibrotischen Pathomechanismen wirksam zu adressieren.

Ergänzend wird eine permissive Anwendung von Protonenpumpenhemmern zur Kontrolle einer gastroösophagealen Refluxsymptomatik empfohlen, da ein möglicher Zusammenhang mit einem günstigeren Verlauf interstitieller Lungenerkrankungen diskutiert wird, wenngleich die Evidenzlage heterogen ist.

## Medizinischer Bedarf bei SARD-ILD

Trotz relevanter Fortschritte in der Behandlung der SARD-ILD mit PPF in den vergangenen Jahren besteht weiterhin ein erheblicher therapeutischer Bedarf. Verfügbare Substanzen können den Verlust der forcierten Vitalkapazität (FVC) zwar verlangsamen, eine Verbesserung des Überlebens wurde in RCTs bisher jedoch nicht nachgewiesen.

So verbesserte CYC in der *Scleroderma Lung Study I* Lungenfunktion und Dyspnoe bei SSc-ILD, Mycophenolat Mofetil (MMF) war in der *Scleroderma Lung Study II *vergleichbar wirksam bei günstigerem Sicherheitsprofil [26, 27]. Nintedanib reduzierte sowohl in der SENSCIS-Studie bei SSc-ILD als auch in der INBUILD-Studie bei progressiven fibrosierenden ILD-Subtypen, einschließlich SARD-ILD, den jährlichen FVC-Verlust signifikant [4, 5]. Für RTX zeigte die RECITAL-Studie in SARD-ILD einen mit CYC vergleichbaren Effekt auf die Verbesserung der Lungenfunktion [16]. Insgesamt belegen diese Studien konsistent eine Verlangsamung der Krankheitsprogression, jedoch ohne nachweisbaren Überlebensvorteil. Auch jüngere RCTs, etwa zu TCZ in SSc-ILD, zu Pirfenidon bei PPF (einschließlich SARD-ILD), zur Kombination Pirfenidon + MMF bei SSc-ILD sowie zu Pirfenidon bei RA-ILD, verfehlten ihre primären Endpunkte, wurden vorzeitig beendet oder waren für harte Endpunkte wie Mortalität nicht gepowert [3, 9, 10, 24].

Darüber hinaus führten verfügbare Antifibrotika trotz nachgewiesener Stabilisierung der Erkrankung bislang weder zu einer relevanten Verbesserung patientenzentrierter Endpunkte noch reduzierten sie die Symptomlast konsistent [25]. Ihr Einsatz ist zudem durch hohe Raten von Nebenwirkungen und Therapieabbrüchen limitiert (z. B. Abbrüche in SENSCIS-ON unter fortgeführter Nintedanib-Therapie 14,7 % vs. 29,1 % unter neu eingeleiteter Nintedanib-Therapie, Abbrüche in Kohortenstudien zur idiopathischen Lungenfibrose [IPF] bis 37,5 % unter Pirfenidon; [1, 17]). Besonders ausgeprägt sind die Verträglichkeitsprobleme bei SSc-ILD, wo gastrointestinale Nebenwirkungen, insbesondere Diarrhoe, die Behandlung erschweren [1, 4].

Pathophysiologisch entsteht SARD-ILD mit PPF durch ein heterogenes Zusammenspiel inflammatorischer und fibrotischer Mechanismen, sodass rein antifibrotische oder rein antientzündliche Therapiestrategien meist unzureichend sind.

Daher besteht ein dringender Bedarf an gut verträglichen, krankheitsmodifizierenden Medikamenten, die sowohl inflammatorische als auch fibrotische Pfade adressieren, dabei die FVC stabilisieren, Symptome lindern, Überlebensraten verbessern und durch ein vorteilhaftes Sicherheitsprofil die Therapietreue steigern und einen frühen Einsatz ermöglichen [11].

Ziel dieses Reviews ist es, aktuelle Entwicklungen und neue Therapieansätze bei SARD-ILD mit PPF darzustellen und deren potenzielle Bedeutung für die klinische Praxis zu bewerten. Hierfür wurde eine systematische Literaturrecherche durchgeführt, die Publikationen bis Mitte 2025 unter den Schlagwörtern „SARD-ILD“,„CTD-ILD“, „PPF“ und „Treatment“ berücksichtigte.

## Nerandomilast als neuartiger Therapieansatz

Nerandomilast stellt als präferentieller Phosphodiesterase-4B-Inhibitor (PDE4Bi) mit immunmodulatorischen, vaskulären und antifibrotischen Eigenschaften einen neuen Behandlungsansatz für die Therapie der SARD-ILD mit PPF dar [14].

PDE4i blockieren den intrazellulären Abbau von zyklischem Adenosinmonophosphat, wodurch die Expression proinflammatorischer Mediatoren reduziert und die Fibroblastenproliferation gehemmt wird [7, 11]. Dies resultiert in kombinierten antiinflammatorischen und antifibrotischen Effekten.

**Abb. 1:**
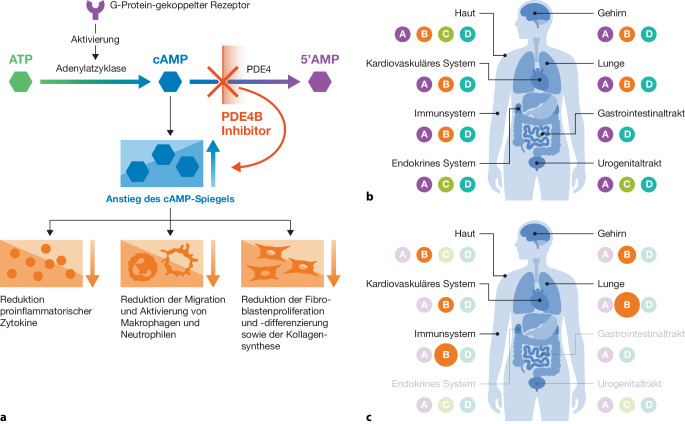
**a** Molekularer Wirkmechanismus eines PDE4B-Inhibitors (mod. nach [7, 11]). **b** Gewebespezifische Verteilung der PDE4-Isoformen im Körper (mod. nach [11]). **c** Gewebespezifische Verteilung der PDE4B-Isoform im Körper (mod. nach [11]). Die Kreisgröße spiegelt die relative PDE4B-Expression und funktionelle Relevanz in den jeweiligen Geweben wider; größeren Kreise repräsentieren eine höhere Expression in Immunzellen und Lungengewebe. *AMP* Adenosinmonophosphat, *ATP* Adenosintriphosphat, *cAMP* zyklisches Adenosinmonophosphat, *PDE4* Phosphodiesterase‑4. (Mit freundl. Genehmigung von © Boehringer Ingelheim Pharma GmbH & Co. KG)

Die Anwendung nichtselektiver panPDE4i wird jedoch durch systemische Nebenwirkungen, insbesondere gastrointestinale Beschwerden, aber auch Kopfschmerzen, Gewichtsverlust und psychiatrische Symptome, stark eingeschränkt [15]. Präferentielle PDE4i wie Nerandomilast, die gezielt den vor allem in Immunzellen und Lungengewebe hoch exprimierten Subtyp PDE4B hemmen und dabei eine etwa 10fach höhere Selektivität für PDE4B gegenüber PDE4D aufweisen, bieten die Möglichkeit, die Wirksamkeit der PDE4i bei deutlich verbessertem Nebenwirkungsprofil zu erhalten (Abb. 1).

Präklinisch zeigte sich unter Nerandomilast eine Hemmung der Fibroblastenproliferation und der Myofibroblastentransformation sowie eine Reduktion des neutrophilen Influx in die bronchoalveoläre Lavage. Außerdem wurden synergistische Effekte mit Nintedanib nachgewiesen [7]. Darüber hinaus zeigte Nerandomilast vasoprotektive Wirkungen sowie in vitro signifikante antifibrotische und antiinflammatorische Effekte in SSc-relevante Zelltypen [22, 23]. Hinweise auf eine Normalisierung pathologischer Transkriptomveränderungen in präklinischen Fibrosemodellen untermauern den Wirkmechanismus von Nerandomilast [21].

## Phase-3-Studie FIBRONEER-ILD

Nerandomilast wurde im globalen FIBRONEER-Programm mit zwei Phase-3-Studien untersucht.

Die doppelblinde, randomisierte, placebokontrollierte Phase-3-Studie FIBRONEER-ILD prüfte Wirksamkeit, Sicherheit und Verträglichkeit von Nerandomilast (9 mg bzw. 18 mg, oral zweimal täglich) bei PPF über ≥ 52 Wochen, stratifiziert nach antifibrotischer Begleittherapie [14].

Nerandomilast erreichte den primären Endpunkt und reduzierte den FVC-Abfall vs. Placebo nach 52 Wochen signifikant (Placebo: −165,8 ml; 9 mg: −84,6 ml [∆ 81,1 ml; 95 % KI 46,0–116,3; *p* < 0,001; ~ 49 %]; 18 mg −98,6 ml [∆ 67,2 ml; 95 % KI 31,9–102,5; *p* < 0,001; ~ 41 %]) und konsistent über vordefinierte Subgruppen (Nintedanib-Begleittherapie, HRCT-Muster und ILD-Diagnosen) hinweg. Beim wichtigsten sekundären Endpunkt (Zeit bis zur ersten akuten Exazerbation, Hospitalisierung aufgrund respiratorischer Ursachen oder Tod) ergab sich vs. Placebo ein numerischer Vorteil für Nerandomilast 9 mg (HR 0,88 [95 % KI 0,68–1,14]) sowie ein deutlicher Trend zugunsten der 18-mg-Dosierung (HR 0,77 [95 % KI 0,59–1,01]). Für das Mortalitätsrisiko zeigten beide Dosierungen einen nominal signifikanten Vorteil vs. Placebo (18 mg: HR 0,48 [95 % KI 0,30–0,79]; 9 mg: HR 0,60 [95 % KI 0,38–0,95]). Zudem wurde für den zusammengesetzten Endpunkt aus Exazerbation oder Tod unter 18 mg eine nominal signifikante Risikoreduktion beobachtet (HR 0,59 [95 % KI 0,41–0,84]), unter 9 mg ein numerischer Vorteil (HR 0,78 [95 % KI 0,56–1,08]).

**Abb. 2:**
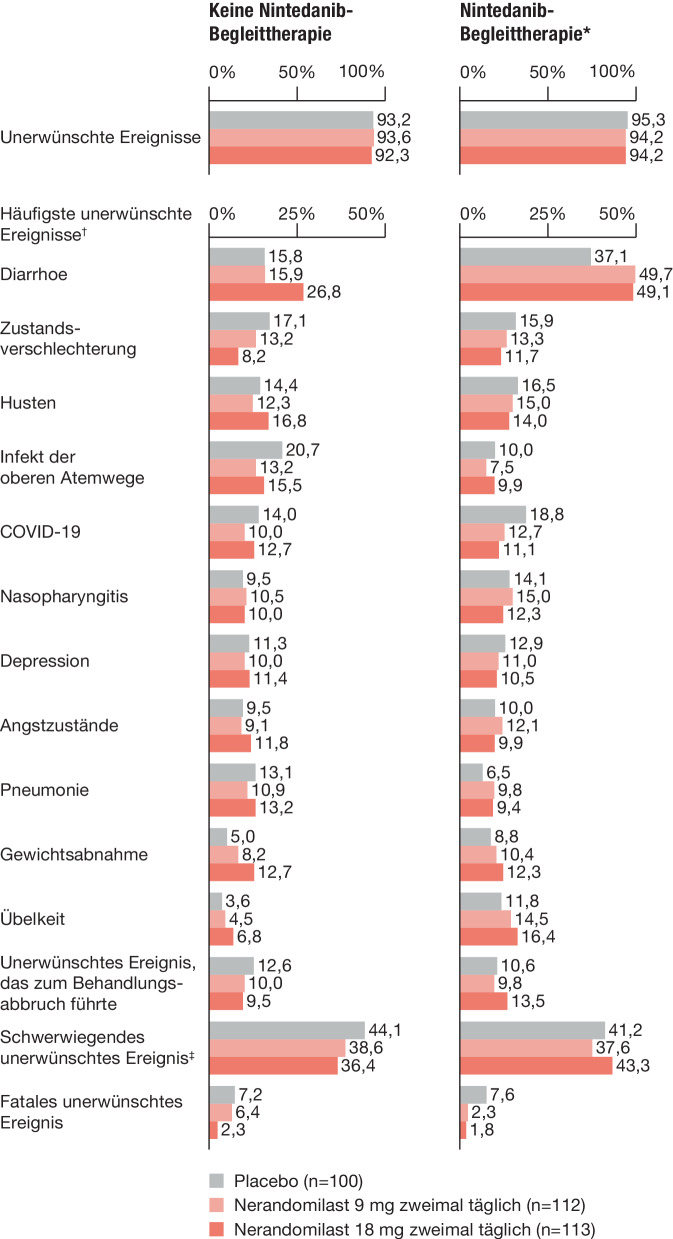
Unerwünschte Ereignisse in der FIBRONEER-ILD-Studie bis zum ersten Database Lock (mod. nach [14]; mit freundl. Genehmigung von © Boehringer Ingelheim Pharma GmbH & Co. KG). *2 Patient:innen dieser Gruppe erhielten Pirfenidon statt Nintedanib. Obwohl die Daten dieser Patient:innen als Protokollabweichungen klassifiziert wurden, wurden sie der Gruppe mit Nintedanib-Begleittherapie zugeordnet und entsprechend analysiert. † Diese Ereignisse wurden anhand der bevorzugten Begriffe des *Medical Dictionary for Regulatory Activities* (MedDRA) kodiert. Dargestellt sind unerwünschte Ereignisse, die bei mehr als 10 % der Patient:innen in einer der Behandlungsgruppen in der Gesamtpopulation auftraten. ‡ Ein schwerwiegendes unerwünschtes Ereignis wurde definiert als ein Ereignis, das zum Tod oder einer Krankenhauseinweisung führte oder eine Verlängerung des Krankenhausaufenthalts erforderte

Nerandomilast wies ein insgesamt günstiges Sicherheits- und Verträglichkeitsprofil auf. Die häufigste Nebenwirkung war Diarrhoe, die Rate an Therapieabbrüchen war vergleichbar mit Placebo (Abb. 2). Das Auftreten weiterer Nebenwirkungen wie Infektionen, arzneimittelinduzierter Leberschädigung, Malignomen und Vaskulitis war zwischen den Behandlungsarmen vergleichbar. Ein Anstieg von Infektionen der oberen Atemwege wurde nicht beobachtet. Therapieabbrüche aufgrund von Nebenwirkungen traten bei Patient:innen mit und ohne Nintedanib-Begleittherapie in ähnlicher Häufigkeit auf.

## Subgruppenanalyse der FIBRONEER-ILD-Studie

**Abb. 3:**
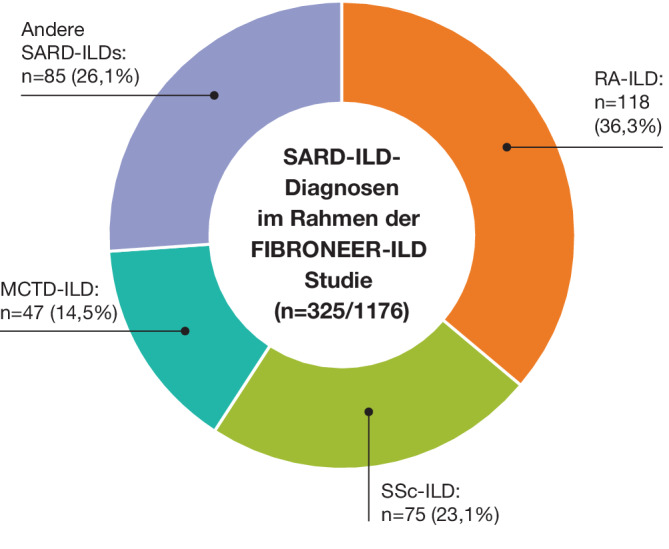
Verteilung der SARD-ILD-Subtypen in der Subgruppenanalyse der FIBRONEER-ILD-Studie (mod. nach [8]). Von den 1176 Teilnehmer:innen der FIBRONEER-ILD-Studie hatten 325 eine SARD-ILD (Placebo: *n* = 100, Nerandomilast 9 mg zweimal täglich: *n* = 112, Nerandomilast 18 mg zweimal täglich: *n* = 113). *ILD* Interstitielle Lungenerkrankung, *MCTD* Mischkollagenose, *RA* Rheumatoide Arthritis, *SARD* Rheumatische Systemerkrankung, *SSc* Systemische Sklerose. (Mit freundl. Genehmigung von © Boehringer Ingelheim Pharma GmbH & Co. KG)

Eine Subgruppenanalyse von FIBRONEER-ILD untersuchte Nerandomilast bei Patient:innen mit SARD-ILD (*n* = 325/1176; Placebo: *n* = 100; 9 mg: *n* = 112; 18 mg: *n* = 113; [8]). Die häufigsten Diagnosen waren RA-ILD, SSc-ILD und Mischkollagenose-assoziierte ILD (MCTD-ILD; Abb. 3).

**Abb. 4:**
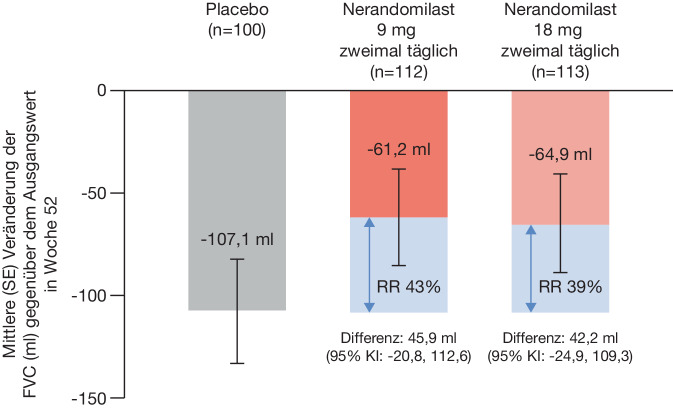
Veränderung der FVC (ml) gegenüber dem Ausgangswert in Woche 52 bei Patient:innen mit SARD-ILD in der Subgruppenanalyse der FIBRONEER-ILD Studie (mod. nach [8]). *FVC* Forcierte Vitalkapazität, *ILD* Interstitielle Lungenerkrankung, *KI* Konfidenzintervall, *RR* Relative Reduktion, *SARD* Rheumatische Systemerkrankung. (Mit freundl. Genehmigung von © Boehringer Ingelheim Pharma GmbH & Co. KG)

Die Wirksamkeit von Nerandomilast auf die Reduktion des FVC-Abfalls nach 52 Wochen entsprach der Gesamtpopulation mit konsistentem Effekt über die verschiedenen Subgruppen (Abb. 4; [8]).

Bezüglich des sekundären Endpunktes (Zeit bis zur ersten akuten Exazerbation, Hospitalisierung aufgrund respiratorischer Ursachen oder Tod) zeigte Nerandomilast 18 mg in der SARD-ILD-Subgruppe einen nominal signifikanten Vorteil (HR 0,56 [95 % KI 0,33–0,96]), Nerandomilast 9 mg einen numerischen Vorteil vs. Placebo (HR 0,71 [95 % KI 0,43–1,20]).

Die Verträglichkeit in der SARD-ILD-Subgruppe war insgesamt günstig. Häufigste Nebenwirkung war Diarrhoe, schwere Nebenwirkungen und therapiebedingte Abbrüche waren vergleichbar mit Placebo.

Mit der derzeit laufenden Phase-3B-Studie FIBRONEER-SARD wird die Wirksamkeit und Sicherheit von Nerandomilast bei SARD-ILD über ≥26 Wochen weiter erforscht. Anders als in FIBRONEER-ILD ist hier eine immunsuppressive Begleitmedikation mit CYC, TCZ, MMF und RTX erlaubt, sodass praxisnahe Daten zum klinischen Alltag generiert werden können. Erstmals werden auch stabile Patient:innen ohne nachgewiesene Progression eingeschlossen, wodurch der Fokus auf präventive Therapieansätze gerichtet wird und ein möglicher Paradigmenwechsel eingeleitet werden könnte.

## Klinische Perspektiven von Nerandomilast

Basierend auf den positiven Studienergebnissen ergeben sich im klinischen Alltag neue Behandlungsoptionen, insbesondere für SARD-ILD-Patient:innen mit PPF, bei denen die Verträglichkeit und die in FIBRONEER-ILD gezeigte Mortalitätsreduktion im Vordergrund stehen. Ebenso könnte Nerandomilast eine therapeutische Option für Personen darstellen, die unter etablierten Antifibrotika starke gastrointestinale Nebenwirkungen oder hepatotoxische Effekte entwickeln und deswegen einen Therapieabbruch erwägen, ein Szenario, das in Real-World-Daten zur IPF sowohl für Pirfenidon (16,6 %) als auch für Nintedanib (16,2 %) beschrieben wurde [12].

Bei Progression trotz Standard-of-Care, einschließlich laufender Nintedanib-Therapie, stellt Nerandomilast eine potenzielle Add-on-Option dar. Die Wirksamkeit war auch unter begleitender Nintedanib-Behandlung konsistent und bietet somit eine Therapieoption bei weiter fortschreitender Fibrose [14]. Für die Gruppe der SARD-ILD mit Krankheitsprogression erscheinen zudem Kombinationen aus Nerandomilast und Immunsuppressiva perspektivisch sinnvoll. Dies wird dadurch unterstützt, dass bereits in FIBRONEER-ILD rund 35 % der Patient:innen Nerandomilast in Kombination mit AZA, MTX oder cDMARDs-Präparate erhielten, während stärker immunsuppressive Wirkstoffe wie CYC, TCZ, MMF und RTX nicht erlaubt waren. Das Sicherheitsprofil von Nerandomilast ist insgesamt entscheidend für die klinische Einordnung. Patient:innen mit fortgeschrittener Lebererkrankung (Child-Pugh A–C) waren von der FIBRONEER-ILD-Studie ausgeschlossen, ansonsten wurden keine relevanten Leberwerterhöhungen beobachtet, ein spezifisches Monitoring scheint somit nicht erforderlich.

Hinweise auf ein Risiko von Aneurysmen oder Arteriendissektionen liegen für Nerandomilast bislang nicht vor und sind auch nicht zu erwarten, da es anders als Nintedanib nicht in die VEGF(Vascular Endothelial Growth Factor)-Signalwege eingreift.

## Weitere zukünftige Therapieansätze bei SARD-ILD

Über die Definition des geeigneten Patient:innenprofils hinaus ist auch die Einordnung von Nerandomilast in den Kontext bisheriger und zukünftiger RCTs von Bedeutung.

Vor dem Hintergrund der bisherigen Therapielandschaft zeigte FIBRONEER-ILD erstmals Hinweise auf eine Mortalitätsreduktion und verlangsamte zudem den FVC-Abfall signifikant und konsistent über vordefinierte Subgruppen hinweg. Zusätzlich ergab sich eine synergistische Wirkung in Kombination mit Nintedanib. Im Kontrast dazu untermauerte die INBUILD-Studie zwar die antifibrotische Wirkung bei PPF, jedoch ohne konsistenten Mortalitätseffekt und mit variierendem Placebo-FVC-Abfall je nach Progressionskriterium [5].

Neben Nerandomilast befinden sich derzeit weitere Substanzen zur Behandlung der SARD-ILD in klinischer Prüfung. Dazu zählen der BLyS-Inhibitor Belimumab, der in Phase-3-Studien bei SARD-ILD und SSc-ILD untersucht wird, sowie der orale Lysophosphatidinsäure(LPA)-Inhibitor Admilparant, der aktuell in einer Phase-3-Studie bei PPF, einschließlich SARD-ILD, geprüft wird. Parallel wird Anifrolumab, ein humaner monoklonaler Antikörper gegen IFNAR1, in einer Phase-3-Studie bei SSc untersucht.

Auch vasomodulatorische Ansätze zeigen Potenzial. So verbesserte das inhalative Prostazyklin-Analogon Treprostinil bei pulmonaler Hypertonie assoziierter ILD (PH-ILD) Belastungstoleranz und FVC [19]. Ähnliche Effekte wurden bei IPF beobachtet, was Rückschlüsse auf das Potenzial bei SARD-ILD erlaubt. Derzeit wird inhalatives Treprostinil in Phase 3 bei PPF evaluiert.

Weitere Immunmodulatoren werden ebenfalls erprobt. Vixarelimab, ein monoklonaler Antikörper gegen den Oncostatin-M-Rezeptor‑β (OSMRβ), wird derzeit in einer multinationalen Phase-2-Studie bei SSc-ILD untersucht. Mit Tezepelumab, einem humanen monoklonalen Antikörper gegen das Zytokin TSLP („thymic stromal lymphopoietin“), adressiert eine Phase-1-Studie erstmals gezielt ein eosinophiles Subkollektiv bei SARD-ILD und PPF.

Zellbasierte Therapien stellen einen weiteren vielversprechenden Therapieansatz bei SARD-ILD dar. Mehrere CAR-T-Zell-Studien befinden sich in frühen klinischen Phasen, darunter RESET-SSc zu CABA-201 in SSc, CD19/BCMA-CAR-T-Zellen bei refraktärer SSc sowie First-in-Human-Studien mit ADI-001, einem Anti-CD20 CAR-T-Zellprodukt auf Basis allogener γδ-T-Zellen.

Neben zellbasierten Therapieansätzen wie der CAR-T-Zell-Therapie gewinnen derzeit auch bispezifische Antikörper zunehmend an Bedeutung. Diese ermöglichen eine simultane Modulation zweier immunologischer Zielstrukturen und könnten perspektivisch eine gezielte immunmodulatorische Therapieoption bei SARD-ILD darstellen.

## Ausblick

SARD-ILD mit PPF sind weiterhin mit einer hohen Krankheitslast und einer ungünstigen Prognose verbunden. Aktuell verfügbare Therapien können die Krankheitsprogression durch antifibrotische Wirkmechanismen lediglich verlangsamen, jedoch nicht aufhalten.

Mit Nerandomilast steht erstmals ein vielversprechender, oraler PDE4Bi kurz vor der Zulassung, der einen Wendepunkt im Management von SARD-ILD mit PPF darstellen könnte, indem er den frühzeitigen und dauerhaften Einsatz gut verträglicher Therapien ermöglicht. Die klinische Relevanz des Wirkstoffs wird dadurch unterstrichen, dass Nerandomilast im Oktober 2025 die Zulassung der US-FDA für die Behandlung der IPF erhielt, gefolgt von der Zulassung in China.

Dennoch bleiben offene Fragen zur Positionierung der Substanz im klinischen Alltag sowie im therapeutischen Algorithmus, etwa zur Verwendung als Add-on bei Progress unter etablierter Therapie, einschließlich laufender Nintedanib-Therapie, oder als frühzeitige Behandlungsoption in einem breiteren Patient:innenkollektiv. Ebenso müssen weitere Daten, insbesondere zu Langzeitverläufen, Real-World-Erfahrungen, Mortalitätsentwicklung sowie Abbruchraten vorliegen, um den Stellenwert der Substanz für die Praxis endgültig zu definieren. Zudem sind Fragen zur Kombination mit antiinflammatorischen Medikamenten von Bedeutung, da diese in der klinischen Praxis häufig eingesetzt werden. Auch bestehen weiterhin Herausforderungen in der Versorgungspraxis, etwa durch Diagnose- oder Therapieverzögerungen sowie das Fehlen verlässlicher Biomarker zur individuellen Verlaufseinschätzung. Ebenso liegen bislang keine belastbaren Daten für Patient:innen mit stark eingeschränkter Lungenfunktion (FVC < 45 %, DLCO < 25 %), zusätzlicher Obstruktion oder CPFE (kombinierte Lungenfibrose mit Emphysem) vor. Außerdem bleibt abzuwarten, wie die Zulassung von Nerandomilast gestaltet wird und welche Patient:innengruppen dadurch abgedeckt werden.

Über Nerandomilast hinaus eröffnen die derzeit laufenden und kommenden Studienprogramme ein vielversprechendes Spektrum innovativer Therapieansätze für die SARD-ILD mit PPF. Mit der Entwicklung neuer, wirksamer und zugleich gut verträglicher Substanzen wächst die Hoffnung, die Prognose von Patient:innen künftig deutlich zu verbessern und Versorgungslücken zu schließen.

## Fazit für die Praxis

- Rheumatische Systemerkrankungen gehen häufig mit einer Lungenbeteiligung einher. Insbesondere SARD-ILD mit PPF sind mit einer hohen Krankheitslast und ungünstigen Prognose verbunden.
- Bestehende Therapien verlangsamen den FVC-Verlust, zeigen jedoch keine konsistente Mortalitätsreduktion und sind oft mit Nebenwirkungen und hohen Abbruchraten assoziiert.
- Es besteht ein hoher Bedarf an gut verträglichen, krankheitsmodifizierenden Therapien, die sowohl inflammatorische als auch fibrotische Prozesse adressieren.
- Nerandomilast, ein präferentieller PDE4Bi mit immunmodulatorischen, vaskulären und antifibrotischen Eigenschaften, verlangsamte in der FIBRONEER-ILD-Studie den FVC-Abfall und senkte das Mortalitätsrisiko bei günstigem Sicherheitsprofil. Dies markiert einen potenziellen Wendepunkt im Management von SARD-ILD mit PPF.
- Weitere laufende Studien zu innovativen Substanzen lassen auf neue therapeutische Optionen und eine bessere Prognose der Betroffenen hoffen.
